# Supplementary material for: Profiling of phytochemicals from aerial parts of Terminalia neotaliala using LC-ESI-MS2 and determination of antioxidant and enzyme inhibition activities
Source: PLoS One. 2022 Mar 31;17(3):e0266094. doi: 10.1371/journal.pone.0266094 (PMC8970405; doi:10.1371/journal.pone.0266094)
Supplement: Attachment — Submitted filename: Rebuttal letter.docx [file pone.0266094.s001.docx]

*PLOS ONE* Journal

plosone@plos.org

October 21, 2021

Subject: **Resubmission of manuscript no. PONE-D-21-22939**

Dear Editors/Reviewers

We acknowledge the time and expertise of editors and reviewers for the technical assessment of our work and generating generous comments on manuscript “Profiling of phytochemicals from aerial parts of *Terminalia neotaliala* using LC-ESI-MS and determination of antioxidant and enzyme inhibition activities”. We have incorporated changes in the manuscript as per recommendation by the reviewers. In the following we address the concerns of editors and reviewers point by point.

We have tried our best to address all the points of editors and reviewers properly and believe that our manuscript has improved considerably.

We look forward to hearing from you soon.

Kind Regards,

Muhammad Nadeem Shahzad

Ph.D. Scholar

Department of Pharmaceutical chemistry

Faculty of Pharmacy,

The Islamia University of Bahawalpur, 63100, Pakistan

On behalf of all authors.

**Reviewer #1:**

The manuscript PONE-D-21-22939 deals with the extraction, antioxidant capacity and enzyme inhibition of Terminalia neotaliala. The authors have also reported the tentative identification of the main phytochemicals of butanol fraction, the most active among the fractions obtained.
I suggest a carefully revision of the manuscript in order to improve the quality and readability of the article.
Attached you will find a highlighted version with including the main comments.

**Response:** The highlighted version of manuscript recommended by reviewer was improved by addressing the main comments and the changes were highlighted yellow.

**Reviewer #2:**

Literature related to the plant, Terminalia neotaliala, are hardly available and the authors made a good choice with regard to plant selection. However, the manuscript lacks coherence, clarity and organization. The following are some of the issues that need careful considerations by the authors.

**Major comments**

**1.** The manuscript is full of technical issues related to language use and hence, requires a throughout revision.

**Response:** The manuscript was checked by all authors for language usage, spelling and grammar and copyedited thoroughly.

**2.** Tables and Figures are wrongly cited throughout the manuscript and are difficult to follow. This in turn reduced the quality of the manuscript.

**Response:** We revised and cited all tables and figures correctly at their proper position throughout the manuscript.

**3.** In text reporting of values should be revised throughout the manuscript. For instance: Line 320-321: "The values ranged from 6.859524±0.218218 mg. GAEq.g-1 DE±SD to 234.7929 ± 0.116934 mg. GAEq.g-1 DE±SD", should be re-written as: "The values ranged from 6.859524 to 234.7929 mg GAEq g-1 DE".

**Response:** The reported values were revised according to reviewer’s recommendation.

**4.** The authors stated that they identified the polyphenols from butanol extract of the plant simply by comparing with previous literature. Such identification process should be explicitly explained using the obtained mass data. Moreover, there are a number of concerns related to the mass data given in Table 3 (should be Table 5). For instance, the elution of quercetin (flavonol) prior to many HHDP-derivative tannins is unexpected in a reverse-phase chromatography. Besides, it's difficult to accurately identify isomeric molecules (as listed in the table) with a mere comparison with literature. Therefore, the authors should support their identification process with additional experiments such as the use available standards. Moreover, they should provide logical explanations with regard to the separation order and fragmentation pattern of each molecule. Together, the authors should provide the LC-chromatogram of the n-butanol extract.

**Response:** LC-MS was employed to simultaneously identify many phenolic constituents separated directly from crude extract and fraction of *T. neotaliala.* The tentative identification of compounds in butanol fraction was based on evaluation of fragmentation pattern of the compounds and their respective mass to charge ratios. Unfortunately, it was not possible for us to perform additional experiments due to non-availability of reference standards.

**5.** The authors claimed that most of the compounds from the n-butanol extract are class of tannin. Thence, total tannin content should be determined along with TPC and TFC.

**Response:** Total tannin content was experimentally determined for *Terminalia neotaliala* extract. The results of tannin quantification were interpreted in terms of mg tannic acid equivalent per gram of dried extract.

**6.** The authors sated that they performed ANOVA (Line 306). Therefore, tables 2, 3 and 4, and their respective in text explanations should reflect statistical data (i.e. the authors should show if the variations observed in contents and biological activities among the different extracts are significant or not).

**Response:** Results for ANOVA have been included in the manuscript.

**7.** What is the difference between Table 3 (antioxidant results) and Figure 1 (Chart plot)? One of them should be removed.

**Response:** Table 3 was removed from the manuscript.

**8.** Figure 2 and Figure 3 legends are misleading. It seems that each of these figures represent specific compounds rather than the whole extract. Besides, it is not clear why the authors provided MS spectrum for specific compounds (Figure 3-7) only.

**Response:** Figure 2 and 3 legends represent LC-ESI-MS spectrum of butanol extract of Terminalia neotaliala in negative ionization mode and positive ionization mode respectively. The authors provided MS spectrum for specific compounds (Figure 3-7) only with a view to not enhance length of manuscript. However, now we have added LC-MS scans of other compounds as well.

**Minor comments:**

**1.** The title can be re-composed as "Profiling of phytochemicals from aerial parts of Terminalia neotaliala using LC-ESI-MS and determination of antioxidant and enzyme inhibition activities", or in another form.

**Response:** This is a very good suggestion. We re-composed the title as per your recommendation.

**2. Section 2.2:** The collected sample should be simply stated as aerial parts.

**Response:** The authors stated the collected sample simply as “aerial parts” in section 2.2 as per reviewer’s recommendation.

**3.** Section 2.4: Amount of solvent and sample, and extraction time should be included.

**Response:** Amount of solvent and sample, and extraction time were included in section 2.4.

**4.** Section 2.9: The LC-condition (including column type, mobile phase, gradient condition, etc) should be clearly stated.

**Response:** We included LC-conditions in section 2.9.

**5.** Some of the sub/section in the Materials & Methods part can be merged.

**Response:** As per your suggestion, procedures for radical scavenging assay and reducing power assays have been merged.

**6.** All the antioxidant assay protocols needs revision and must be clearly written.

**Response:** Antioxidant assay protocols were revised to give detailed description.

**7.** Decimal forms should be uniform while reporting values in tables and texts (better to round values to two decimal places).

**Response:** Decimal forms have been revised in tables and texts.

**Reviewer #3:** This study showed the phytochemical profiling of Terminalia neotaliala by LC-ESI-MS: Antioxidant and enzyme inhibition activity. This paper showed Strong antioxidant activity and presence of high molecular weight hydrolysable tannins by LC-ESI-MS suggest medicinal importance of T. neotaliala. However, it needs the improvement as a whole. Therefore, I would like to suggest to be resubmitted this manuscript.

**1.** Line 120, ‘H. Perrier’ should not be in italic.

**Response:** Font of H. Perrier was changed to non-italic.

**2.** Line 133, 159, revise: Terminalia neotaliala -> T. neotaliala

**Response:** Terminalia neotaliala in Line 133 and Line 159 were replaced with T. neotaliala.

**3.** There were many typo, wrong word, grammar, spacing Etc. I recommended proofreading and correction.
Ex) 450 gm (line 167). Recommended (line 178-179): The obtained fractions were concentrated and dried. The yields 20 g hexane fraction, 120 g chloroform fractioned 90 g butanol fraction. - > The obtained fractions were concentrated and then yielded hexane (20 g), chloroform (120 g), and butanol (90 g), respectively.
IC50 -> IC50 (subscript)

**Response:** The mentioned change has been incorporated in line 178-179 and the whole manuscript was revised and corrected by all authors.

**4.** You should insert number of compounds in respective peaks of chromatogram and increase the resolution

**Response:** The resolution of chromatograms was increased.

**5.** Where is Figure legends?

**Response:** Figure legends have been mentioned in the manuscript at relevant position for figures in the text.

**6.** Recommended edit of all table and figure to show the data well.

**Response:** The tables and figures were edited to present the data well.

**Reviewer #4:** This paper is not recommended to be published in PLOS One because of its simple idea, lack of innovation and insufficient experimental data. Specific problems are as follows:

**1.** The part of Introduction was too much, in which the core content of article was not described enough, but the common sense that was irrelevant to the core idea was heavily described.

**Response:** Introduction was revised in the manuscript.

**2.** The format of full text was not standard, especially the presentation of experimental results including tables and figures.

**Response:** The authors revised the experimental section including tables and figures.

**3.** Table 3 was the same as that expressed in Figure 1. It was unnecessary to repeat the experimental result.

**Response:** Table 3 was removed from the manuscript.

**4.** The position of Figures was not accurately marked in the text.

**Response:** We revised and cited all tables and figures correctly at their proper position throughout the manuscript.

**5.** Though many LC-MS results were provided in this paper, they were not properly analyzed. Moreover, the LC-MS results were so mixed that reviewers could not obtain useful information.

**Response:** LC-MS results were analyzed again and the tentatively identified compounds were arranged in table in the increasing order of their retention times.

**6.** Both ingredient identification and antioxidant activity results were presented in the form of table. The reviewer recommends that the visualized figure is more conducive to the presentation of result. Especially for the identification of ingredients, at least chromatogram result should be provided for reference.

**Response:** We have included figures wherever possible to enhance readability. The chromatograms of all tentative compounds identified from crude extract have been included in the manuscript.

**7.** From above, this paper was not innovative enough. Most of the contents have been reported before, and the contents of independent experiment were insufficient.

**Response:** To the best of our knowledge, other species of genus Terminalia have been explored well but the researchers did not investigate phytochemical composition and biological potential of *Terminalia neotaliala* species.
