# Supplementary material for: Profiling of phytochemicals from aerial parts of Terminalia neotaliala using LC-ESI-MS2 and determination of antioxidant and enzyme inhibition activities
Source: PLoS One. 2022 Mar 31;17(3):e0266094. doi: 10.1371/journal.pone.0266094 (PMC8970405; doi:10.1371/journal.pone.0266094)
Supplement: Attachment — Submitted filename: PONE-D-21-31614 (2021.11.15).docx [file pone.0266094.s002.docx]

This study showed the Phytochemical profiling of *Terminalia neotaliala* by LC-ESI-MS: Antioxidant and enzyme inhibition activity. This paper showed Strong antioxidant activity and presence of high molecular weight hydrolysable tannins by LC-ESI-MS suggest medicinal importance of T. neotaliala. Therefore, I would like to suggest to be accepted this manuscript after the improvement as a whole.

1. Line 54, Total phenolic content (TPC) → total phenolic content (TPC)
2. Line 56, Total tannin content (TTC) → TTC
3. Line 116, 143, 160,….etc. in manusctript, figure , table, *Terminalia neotaliala* → *T. neotaliala*. You did not revise at all. You should write full name (*Terminalia neotaliala*) when you firstly mention the academic name in manuscript, figure, table and then should write as abbreviation (*T. neotaliala*)
4. Table 4, you should check and revise the subscript in formula.
