# Supplementary material for: Profiling of phytochemicals from aerial parts of Terminalia neotaliala using LC-ESI-MS2 and determination of antioxidant and enzyme inhibition activities
Source: PLoS One. 2022 Mar 31;17(3):e0266094. doi: 10.1371/journal.pone.0266094 (PMC8970405; doi:10.1371/journal.pone.0266094)
Supplement: Attachment — Submitted filename: response to reviewers .docx [file pone.0266094.s003.docx]

*PLOS ONE* Journal

plosone@plos.org

January 3, 2022

Subject: **Resubmission of manuscript no. PONE-D-21-22939**

Dear Editors/Reviewers

We acknowledge the time and expertise of editors and reviewers for the technical assessment of our work and generating their comments on manuscript “Profiling of phytochemicals from aerial parts of *Terminalia neotaliala* using LC-ESI-MS and determination of antioxidant and enzyme inhibition activities”. We have incorporated changes in the manuscript as per suggestions by the reviewers. In the following we address the concerns of editors and reviewers point by point.

We have tried our best to address all the points of editors and reviewers properly and believe that our manuscript has improved considerably.

We look forward to hearing from you soon.

Kind Regards,

Muhammad Nadeem Shahzad

Ph.D. Scholar

Department of Pharmaceutical chemistry

Faculty of Pharmacy,

The Islamia University of Bahawalpur, 63100, Pakistan

On behalf of all authors.

**Reviewer #1:**

Reviewer #1: The authors have made huge effort to address previously raised issues and improve the quality of their manuscript for re-submission. However, I still have doubt on the structural identification process. As noted, before, the authors stated that they identified the polyphenols from butanol extract by comparing with previous literature and using LC-MS/MS. I suggested the authors to explicitly describe the fragmentation pattern of each molecule and support their identification process and compound elution order using available standards. However, the authors did not conduct or incorporate any of these in the resubmitted manuscript. They sated that they cannot conduct additional experiment/analysis for unknown reason. Therefore, I still consider that these issues are critical and must be fully addressed for the manuscript to be considered for publication.

**Response:** as per suggestion by the reviewer 1, the manuscript was improved by incorporation of each molecular fragmentation pattern after extensively reviewing the available literature. The quality of each molecular ionogram was also improved. The molecule (132-Hydroxypheophorbide-α-methyl ester) incorporated in table 4, serial # 2 with retention time 5.15 and molecular weight 622 gm/mol belongs to chloroform fraction of *T. neotaliala* which was mistakenly incorporated in butanol fraction (subjected) LC-ESI-MS^2^ molecular table, due to this author were questioned about the order of elution like higher molecular weight eluting before the low molecular weight compounds. Now the mistakenly incorporated misfit compound has been removed from the list of identified compounds. Kindly find the ion-gram of mentioned molecular compound which clearly indicates the chloroform fraction in its description with further details. The authors could not go for further analysis because the LC-ESI-MS^2^ facility was availed from other institute who unfortunately did not have all of the identified standards therefore it’s not feasible for authors to buy each of the standard compounds and run them again due to financial limitations. Therefore, authors have extensively reviewed the previous literature and included the fragmentation patter of each identified compound for supporting the identification process to satisfy the reviewer 1.

**Reviewer #2:**

The manuscript PONE-D-21-31614 was revised for English language and the quality of the presentation and description of the data have been notably improved. The quality of figures 2-22 must be improved and then the work can be accepted and be published in Plos One

**Response:** The quality of English and scientific language has been improved. Similarly, quality of figures 2-21 have been improved at their best quality for possible publication of our manuscript in Plos One.

**Reviewer # 3:**
This study showed the Phytochemical profiling of Terminalia neotaliala by LC-ESI-MS: Antioxidant and enzyme inhibition activity. This paper showed Strong antioxidant activity and presence of high molecular weight hydrolysable tannins by LC-ESI-MS suggest medicinal importance of T. neotaliala. Therefore, I would like to suggest to be accepted this manuscript after the improvement as a whole.

1. Line 54, Total phenolic content (TPC) → total phenolic content (TPC)]
2. Line 56, Total tannin content (TTC) → TTC
3. Line 116, 143, 160,.etc. in manuscript, figure , table, Terminalia neotaliala → T. neotaliala. You did not revise at all. You should write full name (Terminalia neotaliala) when you firstly mention the academic name in manuscript, figure, table and then should write as abbreviation (T. neotaliala)

**Response**

Thanks for the positive comments of the reviewer. The suggested corrections have been made in the revised manuscript.
