# Supplementary material for: Profiling of phytochemicals from aerial parts of Terminalia neotaliala using LC-ESI-MS2 and determination of antioxidant and enzyme inhibition activities
Source: PLoS One. 2022 Mar 31;17(3):e0266094. doi: 10.1371/journal.pone.0266094 (PMC8970405; doi:10.1371/journal.pone.0266094)
Supplement: Attachment — Submitted filename: respone to reviewer 21.docx [file pone.0266094.s004.docx]

**Reviewer #1: (suggestions)**

1. It is preferable to avoid the use of abbreviations while listing keywords.

**Response**

The suggestion has complied while listing keywords

2. please reflect the statistical analysis data in figure 1 (i.e. show if there is any

significant variation in each of the antioxidant activities according to extract types).

**Response**

A statement has been incorporated after statistical analysis

3. Figures are duplicated and hence, need revision.

**Response**

Duplicate figures have been removed

4. The authors have already indicated that the results are reported as mean   SD (lines

306-307). Thence, it is better to avoid the unnecessary repetition of "SD" while

mentioning numerical values in the result and discussion sections (for instance: 234.79

  0.12 mg. GAEq.g-1 DE SD can be rewritten as 234.79   0.12 mg GAEq g-1 DE (line

324), and so forth).

**Response**

The said suggestion has complied throughout the manuscript

5. In the reference section, there is a huge inconsistency in the reference listing.

Authors are advised to follow the journal's guidelines.

**Response**

The suggestion has been made in the bibliography as per Journal guidelines

6. Last but not least, the manuscript still needs a thorough edition and revision towards

its language use (preferably by a native speaker).

Response

The manuscript has been completed edited and revised towards its language
