# Supplementary material for: Profiling of phytochemicals from aerial parts of Terminalia neotaliala using LC-ESI-MS2 and determination of antioxidant and enzyme inhibition activities
Source: PLoS One. 2022 Mar 31;17(3):e0266094. doi: 10.1371/journal.pone.0266094 (PMC8970405; doi:10.1371/journal.pone.0266094)
Supplement: Attachment — Submitted filename: response to reviewer 3 nadeem .docx [file pone.0266094.s005.docx]

**Reviewer #1: Response to comments**

1. The manuscript has been completely revised and edited, toward the English language.
2. The statistical result of figure 1 has been sufficiently described.
